# Demethylation by 5-aza-2'-deoxycytidine in colorectal cancer cells targets genomic DNA whilst promoter CpG island methylation persists

**DOI:** 10.1186/1471-2407-10-366

**Published:** 2010-07-12

**Authors:** David Mossman, Kyu-Tae Kim, Rodney J Scott

**Affiliations:** 1 Discipline of Medical Genetics, School of Biomedical Sciences, Faculty of Health, University of Newcastle, Australia; 2 Hunter Medical Research Institute, NSW, 2305, Australia; 3 Division of Genetics, Hunter Area Pathology Service, John Hunter Hospital, Newcastle, NSW, 2305, Australia

## Abstract

**Background:**

DNA methylation and histone acetylation are epigenetic modifications that act as regulators of gene expression. Aberrant epigenetic gene silencing in tumours is a frequent event, yet the factors which dictate which genes are targeted for inactivation are unknown. DNA methylation and histone acetylation can be modified with the chemical agents 5-aza-2'-deoxycytidine (5-aza-dC) and Trichostatin A (TSA) respectively. The aim of this study was to analyse de-methylation and re-methylation and its affect on gene expression in colorectal cancer cell lines treated with 5-aza-dC alone and in combination with TSA. We also sought to identify methylation patterns associated with long term reactivation of previously silenced genes.

**Method:**

Colorectal cancer cell lines were treated with 5-aza-dC, with and without TSA, to analyse global methylation decreases by High Performance Liquid Chromatography (HPLC). Re-methylation was observed with removal of drug treatments. Expression arrays identified silenced genes with differing patterns of expression after treatment, such as short term reactivation or long term reactivation. Sodium bisulfite sequencing was performed on the CpG island associated with these genes and expression was verified with real time PCR.

**Results:**

Treatment with 5-aza-dC was found to affect genomic methylation and to a lesser extent gene specific methylation. Reactivated genes which remained expressed 10 days post 5-aza-dC treatment featured hypomethylated CpG sites adjacent to the transcription start site (TSS). In contrast, genes with uniformly hypermethylated CpG islands were only temporarily reactivated.

**Conclusion:**

These results imply that 5-aza-dC induces strong de-methylation of the genome and initiates reactivation of transcriptionally inactive genes, but this does not require gene associated CpG island de-methylation to occur. In addition, for three of our selected genes, hypomethylation at the TSS of an epigenetically silenced gene is associated with the long term reversion of gene expression level brought about by alterations in the epigenetic status following 5-aza-dC treatment.

## Background

DNA methylation is an epigenetic modification that occurs on cytosine residues in the sequence context 5'-CG-3'. It is well established that DNA methylation acts as a transcriptional repressor of gene expression via recruitment of repressive proteins. These include the Methyl-CpG Binding Protein 1 (MeCP1) and proteins with a methyl-binding domain, such as MBD1, MBD2, MBD3, MBD4 and MeCP2. These proteins hinder transcription through the recruitment of other factors such as nucleosome remodelling complex [1]. In the case of MeCP2, the protein is capable of binding to a single symmetrically methylated cytosine and contributing to the long-term repression of transcription [2]. The binding of these additional protein factors leads to condensation of DNA and confers stability to the chromosome.

In normal cells, repetitive elements such as long interspersed nucleotide elements, Alu repeats, transposable elements, and satellite and non-satellite sequences which together make up almost half of the genome, are methylated [3-5]. Methylation of these regions largely contributes to the level of global methylation, and it is likely that these regions are most drastically affected by aberrant hypomethylation and the stability that the methylation once conferred to the chromosomes is lost. Aberrant methylation is one of the more frequent molecular changes observed in tumour cells [6] and typically involves the reversal of normal methylation patterns. It has been known for some time that common changes involve genome wide hypomethylation, which impinges on the expression of oncogenes [7], loss of imprinting and hypermethylation of tumour suppressor genes [8]. These are believed to be a cause rather than a consequence of the malignant process as they arise early in disease development [9]. Supporting this is strong evidence that global hypomethylation plays a crucial role in causing genomic instability in colorectal carcinogenesis [10]. Alternatively, gene specific hypermethylation is another mechanism which can initiate carcinogenesis. This mechanism of gene silencing has been shown by the correlation of methylated promoters with a subsequent decrease in corresponding gene expression. The precise set of events that govern which CpG residues are methylated are not understood, nor is the mechanism that causes hypomethylation [11].

5-aza-2'-deoxycytidine (5-aza-dC) is a strong inducer of DNA de-methylation. It is an analogue of cytosine, that when incorporated into DNA, irreversibly binds the methyltransferase enzymes as they attempt to methylate the cytosine analogue. This depletion of methyltransferase in the cell results in passive de-methylation, which is known to reactivate epigenetically silenced genes [12]. 5-aza-dC has demonstrated its most positive effect in the treatment of hematologic malignancy such as myelodysplastic syndromes [13]. In this scenario, its effectiveness may be due to sensitisation to other reagents or the re-activation of silenced genes which have an apoptotic effect. Another agent which affects the epigenetic status of genes is Trichostatin A (TSA). TSA was originally developed as an antifungal agent [14], but was also found to lead to the accumulation of acetylated histones via the inhibition of Histone Deacetylase [15]. The presence of an acetyl group on a lysine amino acid in the N-terminal end of core histone proteins neutralises the positive charge carried by the lysine, weakening the association between the nucleosome and DNA [16] to favour transcriptional activity. Reports have demonstrated a synergistic effect of TSA with 5-aza-dC in the re-expression of epigenetically silenced genes [17-20]. Currently it is not known how methylation patterns are altered with 5-aza-dC, and how or if these patterns can be restored when drug treatment ceases.

The aim of this study was to examine patterns of DNA methylation in several colorectal cancer (CRC) cell lines, to assess how these patterns are affected by drugs which alter epigenetic status, and profile the re-methylation process at both a genome-wide and gene specific level. Currently, the process of re-methylation following 5-aza-dC is not well documented.

## Methods

### Cell Culture

Colorectal cancer cells HCT116, SW48, SW480, HT29 and a fibroblast cell line derived from a healthy donor were all cultured in DMEM supplemented with 10% Foetal Calf Serum (Sigma-Aldrich, St Louis, MO) at 37°C and 5% CO_2_. De-methylation was induced with 5-aza-dC (Sigma-Aldrich) treatment at a pre-determined concentration that induced maximal de-methylation of the DNA without killing the cells. Culture media for LoVo and the fibroblast cells contained 10 μM 5-aza-dC, whilst all other cells were treated with 15 μM. Cells were incubated for 72 h with 5-aza-dC with the culture media being replaced every 24 h with fresh media containing 5-aza-dC. DNA and RNA were extracted before drug treatment and after 72 h of drug treatment. Immediately following drug treatment (72 h), a fraction of the cells were washed twice with PBS and allowed to continue growing under regular drug-free conditions. At every two days following cessation of treatment, DNA and RNA were extracted while a fraction of the cells continued to be incubated until ten days of drug free growth. The experiment was also performed on HCT116 cells continuously exposed to 150 ηM Trichostatin A (Sigma-Aldrich) during the treatment and re-methylation period to assess the affect of histone acetylation on DNA re-methylation. This concentration chosen has previously been shown to cause hyper-acetylation in the HCT116 cell line [21].

### Cytotoxicity and Apoptosis testing

The assessment of cytotoxicity and apoptosis was undertaken using pooled cell cultures as per the assay protocol provided with the detection kits. For practicality, the HCT116, SW480 and LoVo cell lines were assayed. Cytotoxicty was quantified using Cytotoxicty Detection Kit (Roche Diagnostics, Mannheim, Germany) according to manufacturer's instructions. Apoptosis was determined using an Annexin V Apoptosis Detection Kit from BD Biosciences and analysed on a BD FACSCantoII flow cytometer (Becton Dickinson, Franklin Lakes, NJ) following the manufacturer's instructions.

### High Performance Liquid Chromatography (HPLC) Analysis of Global Methylation Levels

50 μg of DNA was treated with 5 μL RNAse Cocktail (Ambion, Austin, TX) to remove any residual RNA which would interfere with HPLC analysis. DNA was then phenol-chloroform extracted and resuspended in sterile water. DNA aliquots of 3 μg were digested with 1.5 U of Nuclease P1 (US Biological, Swampscott, MA) and incubated at 37°C for 16 h. Following digestion, 2 μL of Calf Intestinal Alkaline Phosphatase (Promega, Madison, WI) was added and incubated at 37°C for a further two hours. Separation of nucleosides was performed on a Varian Star Chromatography workstation with a Supelcosil LC-18-DB column (Sigma-Aldrich) over 30 min at 35°C with absorbance monitored at 278 nm. Peak areas were quantified with Star Reviewer Software (Varian, Palo Alto, CA) and the 5-methylcytosine content was expressed as a percentage of the total cytosine pool after correction for extinction co-efficients. Standard deviations were calculated and a T-test was employed to compare expression levels in drug treated cells against untreated cells. *P*-values less than 0.05 were considered to be statistically significant.

### Illumina arrays and Analysis

Illumina Human Ref-8 expression arrays (Illumina, Hayward, CA) were used to measure genome wide gene expression at four chosen time points; untreated cells, treated cells (day 0 of re-methylation), and at four (d4) and ten days (d10) of drug free growth and re-methylation. These time points were selected based on global methylation levels at these time points. Untreated and treated cells were used to identify changes caused by 5-aza-dC, whilst day 4 would allow an intermediate and day 10 a final assessment of the changes in gene expression during re-methylation. Data analysis was performed in Genespring 7.3.1. software (Agilent, Foster City, CA). Genes were classified according to their expression pattern, and were selected with the aim of comparing methylation patterns in genes temporarily reactivated and those still expressed after 10 days of drug free growth. The genes selected for analysis were *CDO1, HSPC105, MAGEA3, RNF113B., ZFP3 *and two colorectal cancer related genes *CDKN2A *and *MLH1*. These genes displayed different expression patterns in the HT29, SW480, SW48 and HCT116 cell lines that could be further examined.

### Bisulfite conversion, PCR and direct sequencing

DNA was converted in duplicate using a Qiagen Epitect Bisulfite conversion kit (Qiagen, Valencia, CA) using 2 μg of phenol-chloroform purified DNA. Samples were eluted in 30 μL of elution buffer and an aliquot was diluted 1:3 prior to PCR and stored at 4°C, whilst the remaining fraction was stored at -20°C. CpG islands surrounding the transcription start site of genes were targeted in PCR analysis using the primers listed in Additional File 1: Table S1. Primers included a non-CpG cytosine at the 3' end in order to preferentially amplify converted DNA sequences. Amplified products were purified with Ampure magnetic bead clean-up system (Agencourt, Beverly, MA). Big Dye Terminator Version 3.1 sequencing mastermix was used with the forward PCR primer and reactions were purified with CleanSEQ magnetic clean-up system (Agencourt). Sequencing was performed in duplicate on an ABI 3730 sequencer and data analysis was carried out using Sequence Scanner software (Applied Biosystems, Foster City, CA). The percentage methylation at each CpG was determined by dividing the cytosine peak by the combined heights of the cytosine and thymine peaks as described previously [22].

### mRNA expression analysis

Extracted RNA was converted to cDNA using Superscript II (Invitrogen, Carlsbad, CA). Real time PCR was performed using an ABI 7500 real time PCR machine and SYBR green mastermix (Applied Biosystems) and primers listed in Additional File 1: Table T1. Reactions were carried out in triplicate and the fold change in expression was normalised to the β-actin housekeeping gene using the 2^-ΔΔCt ^method. For the purpose of calculating fold changes in expression, genes with no detectable expression were assigned a Ct value of 40. Standard deviations were calculated and a T-test was employed to compare expression levels in drug treated cells against untreated cells. *P*-values less than 0.05 were considered to be statistically significant.

### Ethical Approvals

This study was deemed exempt from ethics approval from the University of Newcastle, and consent was not required due to use of cell lines.

## Results

### Global Methylation levels

**Figure 1:**
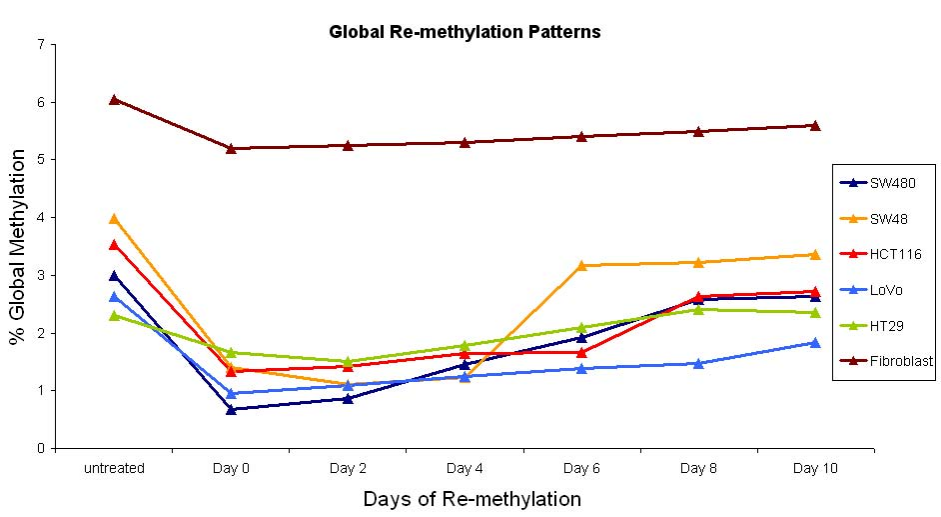
**Global re-methylation in cell lines**. All cells exhibited demethylation after 5-aza-dC treatment, and re-methylation occurred over the 10 days of drug free growth.

Quantitative HPLC indicated that global methylation was lower in all CRC cell lines in comparison to the fibroblast cell line, with a difference of at least 2% in total methylated cytosine (Figure 1). Whilst all cancer cell lines displayed hypomethylation, there were differences in global methylation levels indicating these cells exhibit unique methylation profiles. The cancer cell line HT29 displayed the largest difference in methylation levels compared to the control fibroblast cell line with a total methylated cytosine content difference of 3.75%.

### Global methylation response to 5-aza-dC and re-methylation

Following treatment of cells with 5-aza-dC over 72 hours, there was a substantial decrease of genomic DNA methylation. The decrease in global methylation in the SW48, SW480, HCT116 and LoVo cell lines was greater than 50%, whilst the decrease in fibroblast and HT29 cell lines were not as extensive (Figure 1). Nonetheless, all treated cells had significantly lower levels of methylation compared to untreated cells (p < 0.01). By the tenth day of drug free growth, global methylation levels approached those observed prior to drug treatment, and the HT29 cell line had reached pre-treatment levels by Day 8. This observation suggests there is remodelling of the chromatin state. Cytotoxicity was monitored and found to be elevated following drug treatment, which subsequently receded as growth was continued in drug-free media (Additional File 2: Figure S2). Similarly, an increase in apoptosis was induced by 5-aza-dC exposure, which gradually diminished during the drug free growth period (Table 1).

**Table 1:** Cell Death induced by 5-aza-dC and during recovery period.

Cell Line	Time Point	Viable (%)	Necrotic (%)	Apoptotic (%)
**HCT116**	Untreated	94.6	5.2	0.2
	5-aza-dC treated	94.0	5.5	0.5
	4 d post treatment	89.8	9.9	0.3
	10 d post treatment	94.0	5.6	0.4
**SW480**	Untreated	99.8	0.1	0.1
	5-aza-dC treated	80.3	14.2	5.5
	4 d post treatment	90.5	8.7	1.4
	10 d post treatment	92.6	6.2	1.2
**LoVo**	Untreated	78.4	9.4	12.2
	5-aza-dC treated	67.2	7.0	25.8
	4 d post treatment	67.0	10.7	22.3
	10 d post treatment	77.6	11.3	11.1

Necrosis and cells undergoing apoptosis increased after 5-aza-dC exposure. The number of necrotic cells remained elevated ten days post treatment in SW480 and LoVo whilst apoptosis was decreasing in all cell lines ten days post treatment.

### Global methylation response to combined 5-aza-dC and TSA and re-methylation

Combined TSA and 5-aza-dC treatment was performed on the HCT116 cell line to observe whether histone acetylation would influence the process of DNA re-methylation. Measurement of genomic methyl-cytosine levels revealed no synergistic effect of combined TSA and 5-aza-dC treatment on the de-methylation and no influence of acetylated histones on the re-methylation process in these cells (Table 2).

**Table 2:** Genomic methylation of cells treated with 5-aza-dC alone and in combination with TSA.

Time after 5-aza-dC	5-aza-dC & TSA	5-aza-dC alone	p-value
**day 0**	1.52%	1.18%	0.06
**day 2**	1.55%	1.52%	0.82
**day 4**	2.29%	2.32%	0.60
**day 6**	2.29%	2.37%	0.14
**day 8**	2.31%	2.38%	0.31
**day 10**	2.41%	2.39%	0.76

Global methylation levels decreased with 5-aza-dC treatment and were gradually restored. T-test p-values indicate there is no synergistic effect caused by histone acetylation in the demethylation of genomic DNA.

### Gene-expression analysis

Numerous silenced genes were reactivated after 72 h treatment with 5-aza-dC in each cell line as determined by Illumina expression microarrays. In the cancer cell lines, eight genes were commonly reactivated (Table 3) and a greater number of these remained expressed 10 d post treatment, whilst more short term reactivation was observed in the fibroblasts (Table 4). Hyper-acetylation induced by TSA in HCT116 cells resulted in an increased number of long term and fewer short term reactivated genes compared with 5-aza-dC treatment alone. Of the 511 genes temporarily reactivated with 5-aza-dC in HCT116 cells, 165 were re-expressed in enduring manner when also subjected to 5-aza-dC and TSA combination treatment, suggesting a role of histone acetylation in long term reactivation.

**Table 3:** Commonly reactivated genes in 5-aza-dC treated colorectal cancer cells

**Accession No**.	Gene Name
NM_203339.1	Clusterin
NM_176791.3	Gametocyte specific factor 1-like
NM_173357.2	Synovial sarcoma, × breakpoint 6 (Pseudogene)
NM_144701.2	Interleukin-23 receptor
NM_080618.2	CCCTC-binding factor (zinc finger protein)-like
NM_032598.3	Spermatogenesis associated 22
NM_012253.2	Transkelotase-like 1
NM_006001.1	Tubulin alpha 3C

Eight genes were commonly reactivated across the five cell lines exposed to 5-aza-dC for 72 h.

**Table 4:** Expression patterns of reactivated genes.

Cell Line	Short Term	Long Term	Other
**Fibroblasts**	626	453	356
**HCT116**	511	702	244
**HCT116 + TSA**	354	943	134
**SW480**	383	650	246
**SW48**	525	778	248
**HT29** **LoVo**	551 1116	670 597	257 185

Short term genes were transiently reactivated with 5-aza-dC whilst long term genes were still expressed 10 d post treatment. Other refers to any pattern other than short or long term re-expression.

**Figure 2:**
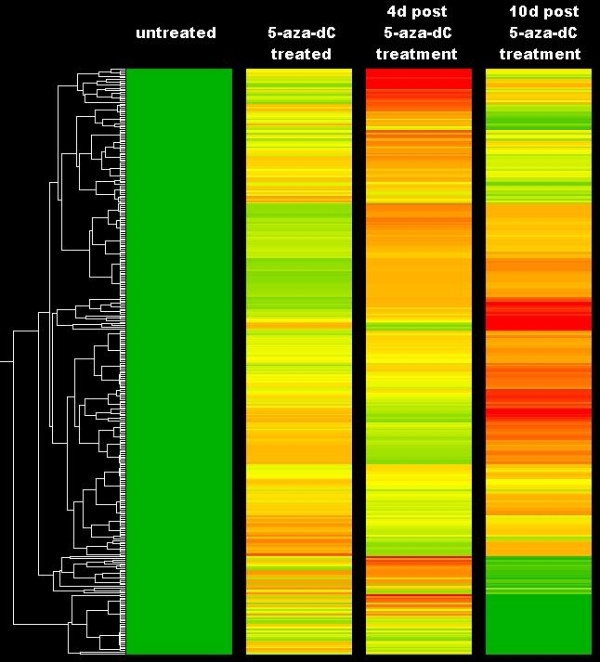
**Selection of genes for bisulphite sequencing analysis**. Shown in the heatmap are the genes reactivated in the SW480 cell line. Filters were applied to select silenced genes that were reactivated upon 5-aza-dC treatment. Genes demonstrating differential expression between cell lines were chosen for further analysis.

Differences were observed in the resulting level of expression of reactivated genes between cell lines in the days following removal of 5-aza-dC. Filtering of the data allowed categorization of two groups of reactivated genes; those that remained highly expressed 10 d post treatment, and those which reverted to an inactive or lowly expressed state (Figure 2). Lists of genes which were expressed according to these opposing groups in two different cells lines were generated. Genes associated with a CpG island were randomly selected for bisulphite sequencing analysis to allow comparisons between long and short term reactivated genes.

### Gene methylation and expression in response to 5-aza-dC

Bisulfite sequencing of seven individual gene promoter regions revealed that de-methylation and re-methylation changes observed at the genome-wide level were not reflected in CpG Island (CGI) methylation levels of the seven genes examined. Global levels decreased by over 50% in some cell lines, however the reduction of methylation at specific gene CGIs was significantly less. The highest de-methylation observed at a specific gene was ~25% at the *RNF113B *CGI in the HT29 (See Additional File 3: Figure S3), SW480 and HCT116 cell lines.

**Figure 3:**
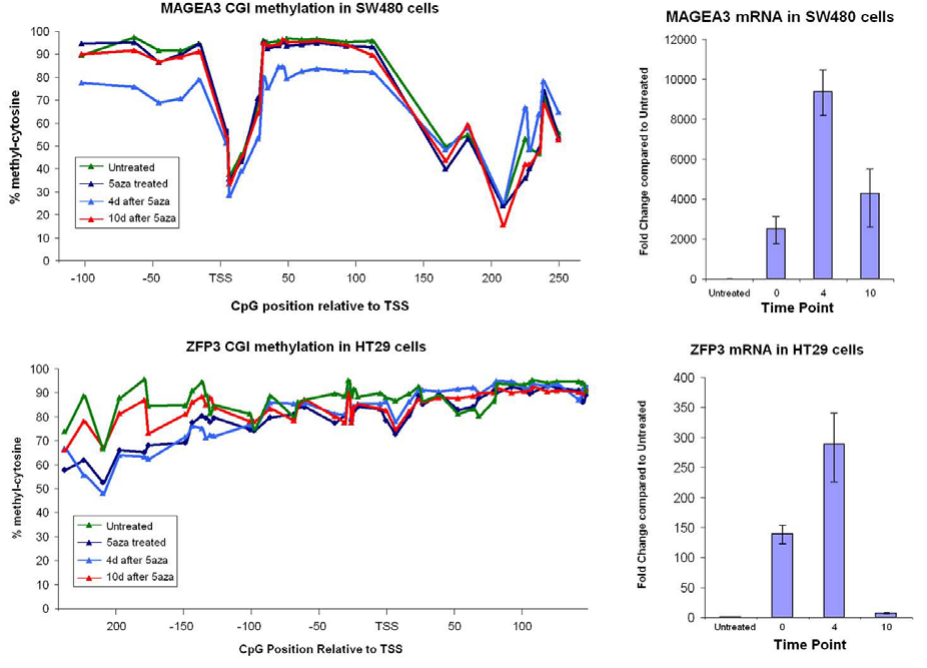
**MAGEA3 and ZFP3 CGI methylation and expression with 5-aza-dC treatment**. Figure 3A illustrates hypomethylated cytosines at the TSS in otherwise hypermethylated CpG island and the corresponding expression (B). Figure C and D shows CGI hyper-methylation and expression of the temporarily reactivated ZFP3 gene in HT29 cells.

Up to 400 bp of sequence data was analysed by bisulfite sequencing of the CGIs surrounding the Transcription Start Site and where possible, the ATG start codon of the respective gene. Several genes revealed the presence of hypomethylated cytosines within an otherwise hypermethylated CGI, and frequently the hypomethylated cytosines would lie in close proximity to the TSS. Genes demonstrating this feature were the *MAGEA3 *(NM_005362.3) (Figure 3), *CDO1 *(NM_001801.2) and *HSPC105 *(NM_145168.2) in SW480 cells, *MAGEA3 *and *CDO1 *in HT29 cells (Additional File 4: Figure S4) and *MLH1 *(NM_000249.2) in SW48 cells (Additional File 5: Figure S5). These genes all appeared to be epigenetically silenced in untreated cells and were reactivated and highly expressed 10 days after 5-aza-dC removal.

Partial methylation was detected in the CGI of *MAGEA3 *and *HSPC105 *genes that were actively transcribed in untreated cells however no methylation was detected in the CGI associated with the beta-actin housekeeping gene in any of the cell lines tested. The *MAGEA3 *and *HSPC105 *genes in HCT116 and HT29 cells respectively, were both expressed and with CGI methylation of ~50-60% suggesting only one allele was hypermethylated.

HCT116 and SW48 cells are of particular interest since the hypermethylation of the promoter regions of *CDKN2A *(NM_000077.3) and *MLH1 *respectively are considered to result in the repression of these genes' expression. In the HCT116 cells, the *CDKN2A *CGI displayed a region of 50% methylation in the vicinity of the TSS that spanned at least 100 bp (Additional File 6: Figure S6). Consequently, *CDKN2A *expression was detected at all times points in these cells. With the exception of SW48, *MLH1 *was expressed in all cells, and no methylation was detected in MLH1 CGI. After 10 days of drug free growth, *MLH1 *was still expressed in SW48 cells, but this did not correlate with the methylation status of the *MLH1 *CpG island, which was hypermethylated.

While some genes were still highly expressed 10 days after 5-aza-dC treatment, there were a group of genes which were temporarily up-regulated or re-expressed but were expressed at low levels after 10 days of drug free growth. Genes in this category that were studied include the *ZFP3 *(NM_153018.1) (Figure 3) and *RNF113B *(NM_178861.3) genes in the HT29 cell line. De-methylation was observed at the *RNF113B *CGI after 5-aza-dC exposure and this level returned to normal levels 10 days after removal of the 5-aza-dC. Genes classified as temporarily reactivated by 5-aza-dC had uniform levels of hypermethylation, unlike those classified as 'permanently' reactivated which carried hypomethylated CpG sites.

## Discussion and Conclusions

The involvement of epigenetic factors, particularly DNA methylation on the regulation of gene expression has been recognised for quite some time, however it is a process not totally understood. The events which underlie genomic hypomethylation and hypermethylation of tumour suppressor genes in malignant cells and why these types of genes are targeted remain unresolved. HPLC analysis of genomic DNA was performed to assess the level of total methylation content present in several types of CRC cells. Genome wide hypomethylation was observed in all the CRC cell lines analysed when compared with the fibroblast cell line, and the lower levels of global methylation in the LoVo and HT29 cell lines may contribute to the genomic instability observed in these cell lines. This finding is consistent with previous reports that malignant cells have lower levels of genomic DNA methylation when compared with healthy tissues [23,24]. The de-methylation observed on a global level did not correlate with that found at specific CGIs, and consequently, expression was observed from genes associated with a hypermethylated promoter. The discrepancy between global and specific gene methylation levels can be explained by the reduced methylation level of Alu elements and LINE methylation. Yang and colleagues [25] demonstrated Alu elements and LINE methylation is reduced by 16% and 60% respectively in colorectal cancer cell lines treated for 72 h with 5-aza-dC. Repetitive regions are likely to fall within compacted heterochromatin where methyltransferase access to the DNA is limited and as a result these sequences become more readily hypomethylated. Furthermore, methylation reductions in a combination of transposable elements [5], satellite repeats [26] and other methylated GC rich areas of the genome which do not form *bona fide *CpG islands [27] may also contribute to this difference.

### Global methylation levels and response to 5-aza-dC and TSA

In all cell lines studied significant genome-wide de-methylation was observed, and a greater than 50% reduction was observed in SW48, SW480 and HCT116 cells, indicating significant DNA de-methylation. Amongst the cancer cells, higher pre-treatment levels of global methylation appeared to correlate with a larger decrease in global methylation levels. Even with exposure to a high concentration of 5-aza-dC, global methylation levels following treatment averaged ~1.2% in the colorectal cancer cell lines. This may be interpreted to suggest that if total methylation levels fall below this point, cell death may be induced either through de-methylation, or toxicity from excessive DNMT binding to DNA. Further analysis of DNA with genome wide methylation arrays might reveal regions that are common to all cell lines which are resistant to DNA de-methylation induced by 5-aza-dC. This possibility was not examined in the current study.

By the tenth day of drug free growth, the global methylation of all cell lines had increased and was nearing pre-treatment levels. The rate of re-methylation was steady in the SW480, LoVo, HT29 and fibroblast cell lines, whilst periods of rapid re-methylation were observed in HCT116 and SW48 cell lines (Figure 1). Despite genomic methylation increasing, the rate was reduced dramatically after this point which may suggest levels have, or could, plateau below the original level. This scenario could be indicative of an altered pattern of gene expression following 5-aza-dC treatment. The HCT116 and SW48 cell lines are known to harbour epigenetic changes, and were among the most affected by 5-aza-dC which may suggest regulation of DNA methylation in these cell lines is different from the others studied.

Continual exposure of the HCT116 cells to Trichostatin A was performed in combination with regular 5-aza-dC treatment to investigate whether altering histone acetylation and chromatin conformation could affect the DNA methylation process and subsequent gene expression patterns. TSA is an inhibitor of Histone Deacetylase and leads to histone hyper-acetylation. Histone acetylation is associated with regions of active chromatin [28] and has been shown to assist the binding of a the TFIIIA transcription factor to chromatin templates [29]. With this knowledge, an investigation into whether global de-methylation could be enhanced and if re-methylation could be restricted by histone hyper-acetylation was undertaken. Our results show that at a genome wide level, TSA did not enhance the de-methylation process in HCT116 cells, and continual exposure to TSA for ten days did not significantly alter the re-methylation process (Table 2) in the HCT116 cell line. The p-value of 0.06 indicates the largest difference is at the d0 time point, however at this time the TSA treated cell line had a greater level of global methylation, and therefore did not enhance de-methylation. Based on these observations, DNA methylation is not hindered by histone acetylation. This notion does not conflict with previous findings that 5-aza-dC and TSA have a synergistic effect on gene expression [17,30] rather it indicates methyltransferase enzymes are not deterred from hyper-acetylated DNA.

Expression arrays were conducted with the aim of identifying reactivated genes that were differentially expressed between the cell lines and could be subjected to bisulfite sequencing analysis. In addition, the influence of TSA on expression patterns could be observed. Combined treatment of TSA with 5-aza-dC did not cause an increase in the number of reactivated genes which is in accordance with its minor influence on genomic methylation levels. Again, this does not conflict with previous reports of a synergistic effect of the two drugs, but indicates DNA methylation plays a greater role than histone acetylation in reactivating silenced genes on a genome wide level [20]. The synergistic effect of TSA was observed with prolonged expression of 165 genes deemed temporarily reactivated with 5-aza-dC alone, implicating TSA treatment as a valuable tool for maintaining expression of genes reactivated with 5-aza-dC.

The combined use of 5-aza-dC and TSA may be advantageous in overcoming poor outcomes in tumour types that do not respond to 5-aza-dC alone. A 'maintenance' administration of TSA following a 5-aza-dC treatment cycle may assist with prolonged gene expression without the cytotoxic effects of 5-aza-dC. Furthermore, identification of the pre-existing methylation patterns at genes targeted for reactivation could determine whether that gene will respond to treatment and whether a particular patient is suitable for this type of therapy.

### Bisulphite sequencing of CpG islands

Bisulphite conversion of DNA followed by PCR and direct sequencing across a CGI permits quantification of the methylation at individual CpG sites and allows for the establishment of a methylation profile of CpG islands. The genes studied displayed varying levels of responsiveness to 5-aza-dC treatment, as observed by a decreased CGI methylation, however the decrease was not consistent with that observed on a genome wide level. The largest decrease in gene specific methylation was in the *RNF113B *gene. After treatment, CGI methylation levels dropped by over 20% in the HT29 cell line with an associated increase in gene expression. By the tenth day of drug-free growth the CGI methylation returned to pre-treatment levels, which correlated with the return of normal levels of gene expression.

Sequencing of CGIs allowed the detection of several instances where a small cluster of cytosines were hypomethylated amongst an otherwise hypermethylated CpG island in a non-expressed gene. These hypomethylated cytosines appear at a CpG site adjacent to the TSS of a gene, as seen in the *MAGEA3 *CGI in SW480 cells. Upon culturing the cells for a further four and ten days in drug free media, these cells were found to still be expressing the previously silenced *MAGEA3 *gene, suggesting the transcriptional status of this gene had been permanently reversed. In comparison, the HCT116 cell line expressed these two genes constitutively. The only common methylation pattern amongst the two cell lines was <50% methylation at the CpG sites 1 bp upstream and 10 bp downstream of the TSS in the *MAGEA3 *gene. This kind of reactivation was also observed in *CDO1*, and *HSPC105 *genes in SW480 cells which also carried hypomethylated CpG sites.

To investigate the possibility that the enduring reactivation of genes was due to continued cytotoxicity or apoptosis, we monitored these levels over the corresponding time period. Both cytotoxicity and apoptosis levels were elevated by 5-aza-dC, but decreased when the drug was removed. This indicates gene expression variation is a result of changes in genomic methylation rather than activation of apoptotic pathways. The fraction of necrotic cells remained higher than in untreated cells which are likely to represent reactivated genes that orchestrate cell death or cells that died in the time prior to the measurement of apoptosis.

Treatment of cells with 5-aza-dC caused the reactivation of numerous genes, although de-methylation within the CGI of specific genes did not correlate with genomic levels (as discussed earlier) or transcription levels. Consequently we observed that 5-aza-dC induced expression can be driven from a largely methylated promoter with localised demethylation at the TSS. One such example is the *CDO1 *gene in SW480 cells (Additional File 7: Figure S7), where ~10% de-methylation (ie 10% of all alleles) at three CpG sites caused a greater than 1000-fold up-regulation of CDO1 mRNA.

We envisage the significant increase in expression of CDO1 is inflated due to non-existent expression in untreated cells, and that modest expression is permitted due to hypomethylation in a small proportion of cells at the TSS. As methylation of surrounding CpG sites was largely unaltered, demethylation of three CpG sites in a 50 bp region surrounding the TSS is permissive of transcription, and does not require hypomethylation of the entire allele. A similar finding has been previously reported in *CDKN2A *in cervical carcinogenesis [31] and also in the *CDH1 *gene in [32]. The minor demethylation of *CDH1 *in conjunction with large increase of gene expression may suggest that DNA methylation does not repress transcription, and gene up-regulation could be due to either a secondary effect or the involvement of other factors which are also modified by 5-aza-dC treatment.

Clusters of CpG sites were identified in some genes that showed a region of hypomethylation, such as *MAGEA3 *in HT29 cells (Additional File 4: Figure S4) and *ZFP3 *in the SW480 cells (Additional File 8: Figure S8). Expression of these genes was detected before 5-aza-dC treatment, demonstrating the importance of methylation in the CpG sites around the TSS, and how a region of hypomethylation is permissive of transcription. Methylation of ~50% was detected at some CGIs including *CDKN2A *in HCT116 cells. These genes are likely to show mono-allelic methylation which has been reported previously [33,34], where all expression is from one non-methylated allele.

The *MLH1 *gene is silenced by methylation on both alleles in SW48 cells [35]. Following 5-aza-dC treatment, de-methylation was induced and the gene was re-expressed and by the tenth day of drug free growth, the gene was still expressed despite methylation returning to pre-treatment levels. The expression level of *MLH1 *at day 10 may be due to lower methylation at a CpG site 32 bp downstream from the TSS, which is similar to the long term reactivation in the *CDO1*, *MAGEA3 *and *HSPC105 *genes in SW480 cells. The analysis undertaken suggests that genes thought to be under control of epigenetic modifications such as *CDKN2A *and *MLH1 *were not shown to have significantly altered patterns of CpG methylation following 5-aza-dC treatment, although an increase in expression was observed.

In non-expressed genes, the identification of regions of hypomethylated cytosine in a generally hypermethylated CpG island raises the question of how these cytosines are maintained in a hypomethylated state. As the region of hypomethylated cytosines in these genes is less than 146 bp - the length of DNA associated with a nucleosome, it would suggest in this scenario at least, an absent nucleosome is not a factor in assisting transcription as previously described [36]. It would appear 5-aza-dC can induce an irregular nucleosomal conformation that permits expression from methylated genes. It is also possible that 5-aza-dC reduces repressive histone tail marks or initiates the gain of active marks in the area surrounding the hypomethylated cytosine following 5-aza-dC exposure. The methylation of Histone H3 Lysine 9 is a modification associated with inactive chromatin and has been shown to be rapidly lost with 5-aza-dC treatment [37]. It is possible that a loss of H3K9 methylation may have been induced by 5-aza-dC in the current study, which may allow binding of transcriptional proteins, which in turn enhance transcriptional reactivation.

Hypomethylated CpG sites which exert control on gene expression may have implications for methods such as methylation-specific PCR and array technologies which rely on the methylation status of a small number of CpG sites in order to determine a given genes methylation status. The observation that a single or small group of CpG sites could affect expression may have greater implications should a polymorphism exist at the site. A polymorphism at the CG dinucleotide will deny methyl-group attachment which would be advantageous to individuals with that change by conferring a protective effect against epigenetic inactivation, particularly in genes such as *MLH1*.

Our results show that 5-aza-dC induces gene expression, but is not necessarily dependant on DNA de-methylation. The pre-existing level of methylation surrounding the transcriptional start site of a gene appears important in long term reactivation. This was demonstrated as the transcriptional status of silenced genes could be reversed with 5-aza-dC and for up to ten days after its removal in genes with hypomethylated cytosines despite minimal CpG de-methylation. In CGIs which exhibited a uniform level of hypermethylation, transcription could be induced for a short term only. The acetylation of histones was not found to alter the de-methylation or re-methylation process and was therefore not expected to cause a change in the gene expression profile. Although constitutively expressed genes demonstrate hypomethylation, 5-aza-dC treatment was found to force expression from genes with hypermethylated CGIs, suggesting that 5-aza-dC is capable of influencing other factors involved with gene expression, such as proteins with a methyl-binding domain or histone modifications.

## Abbreviations

CGI: CpG Island; CRC: Colorectal Cancer; DNA: deoxyribonucleic acid; 5-aza-dC: 5-aza-2'-deoxycytidine; HPLC: High Performance Liquid Chromatography; MeCP: Methyl-CpG binding Protein; PBS: Phosphate Buffered Saline; PCR: Polymerase Chain Reaction; RNA: ribonucleic acid; TSA: Trichostatin A; TSS: Transcription Start Site.

## Competing interests

The authors declare that they have no competing interests.

## Authors' contributions

DM performed the HPLC, microarray, bisulfite sequencing assays, analysis and drafted the manuscript. KTK performed the cytotoxicity and cell survival assays. RJS conceived the study, and participated in its design and co-ordination. All authors have read and approved the final manuscript.

## Pre-publication history

The pre-publication history for this paper can be accessed here:

http://www.biomedcentral.com/1471-2407/10/366/prepub

## Supplementary Material

Additional file 1 **Table S1. Primer sequences used in Bisulfite PCR/Sequencing and qPCR**. Click here for file

Additional file 2 **Figure S2. 5-aza-dC induced cytotoxicity levels and recovery**. Cytotoxicity was elevated immediately following treatment. By day 10 of the recovery period these levels had subsided to at least half of the initial value. Click here for file

Additional file 3 **Figure S3. RNF113B methylation in HT29 cells**. The RNF113B gene is methylated (A) and lowly expressed (B) in HT29 cells. 5-aza-dC induces de-methylation of up to 30% which corresponds with an increase in expression of the gene. Methylation of the RNF113B CGI and expression at day 10 approach levels observed in untreated cells. Click here for file

Additional file 4 **Figure S4. MAGEA3 and CDO1B CGI methylation in HT29 cells**. Both of these CGIs demonstrate hypomethylation at the TSS (A and C). Following 5-aza-dC treatment both genes were still expressed after 10 days of drug free growth (B and D). Click here for file

Additional file 5 **Figure S5. MLH1 CGI methylation in SW48 cells**. The methylation of SW48 does not change dramatically with 5-aza-dC treatment (A), but expression is reactivated and remains high after ten days of drug free growth (B). Click here for file

Additional file 6 **Figure S6. CDKN2A CGI methylation in HCT116 cells**. Methylation of the CDKN2A CGI is approximately 50% surrounding the TSS (A). Expression is detected in untreated cells and becomes up-regulated after 5-aza-dC treatment (B). Click here for file

Additional file 7 **Figure S7. CDO1 CGI methylation in SW480 cells**. A decrease of ~10% of promter methylation in the CDO1 promoter region results in an 1000 fold increase in expression. Click here for file

Additional file 8 **Figure S8. ZFP3 CGI methylation in SW480 cells**. A cluster of hypomethylated cytosines are present at the TSS but a greater level of methylation is observed adjacent to this region. Expression is up-regulated and remains high after ten days of drug free growth. Click here for file
