# Supplementary material for: Demethylation by 5-aza-2'-deoxycytidine in colorectal cancer cells targets genomic DNA whilst promoter CpG island methylation persists
Source: BMC Cancer. 2010 Jul 12;10:366. doi: 10.1186/1471-2407-10-366 (PMC2912869; doi:10.1186/1471-2407-10-366)
Supplement: Additional file 1 — Table S1. Primer sequences used in Bisulfite PCR/Sequencing and qPCR. [file 1471-2407-10-366-S1.DOC]

| **Gene**  **(Accession Number)** | **Bisulfite PCR and Sequencing (5’-3’)** | **qPCR (5’-3’)** |
| --- | --- | --- |
| **GAPDH (NM_002046.3)** | F: GTTGGGATTGGTTGAGTT | n/a |
|  | R: CCAAACCTCCATACCCAAC |  |
| **ACTB (NM_001101.2)** | n/a | F: TGTGGCATCCACGAA ACTACC |
|  |  | R: ACATCTGCTGGAAGGTGGACA |
| **CDO1 (NM_001801.2)** | F: TTAAAGTGGGGGAGAGATTG | F: GAGGGAAAACCAGTGTGCCTAC |
|  | R: AACCTACACCTCCTCTACATTA | R: GCTCACAGCAGGTTCCGTATG |
| **HSPC105 (NM_145168.2)** | F: GTGAAAGTTTAAAAGTAGATAT | F: GTGTCCTCATTACAGGAGG |
|  | R: CATTCTAAAAAACCAAACTAC | R: GCTTTCTCTACGTCAGACAGG |
| **MAGEA3 (NM_005362.3)** | F: GGATTTATAGTTTTAGGAT | F: ATCTGCCAGTGGGTCTCCATT |
|  | R: CACATTAAACTCTATCCCCAAAA | R: TCTGCTCAAGAGGCATGATGA |
| **RNF113B (NM_178861.3)** | F: GGTTAGGTTGGTTTTAAATTGTTGATT | F: GTGTTTCATATGTCGCCAGGCC |
|  | R: CTAAAACCTACAACCCCTTTC | R: CGGTTGGCTGGTCACAGATG |
| **ZFP3 (NM_153018.1)** | F: GAGTTTTTGAGTTTAGAGTAATGT | F: CTTCGGGCAGAGTTCTGAGC |
|  | R: CATAAACTTCAAAATCACACAAC | R: CTGAGTTCCCCCTGAAGGCC |
| **CDKN2A (NM_000077.3)** | F: GATTTTAGGGGTGTTAT | F: GTCGGAGGCCGATCCAGGTCATG |
|  | R: CTCATTCCTCTTCCTTAAC | R: AGCGTGTCCAGGAAGCCCTC |
| **MLH1 (NM_000249.2)** | F: AGATTATTTTAGTAGAGG | F: AGCTGATGGAAAGTGTGCATACA |
|  | R: AAAAAACCTAACTAACA | R: CGTGATCTGGGTCCCTTGA |
