# Supplementary figures and images for: Demethylation by 5-aza-2'-deoxycytidine in colorectal cancer cells targets genomic DNA whilst promoter CpG island methylation persists
Source: BMC Cancer. 2010 Jul 12;10:366. doi: 10.1186/1471-2407-10-366 (PMC2912869; doi:10.1186/1471-2407-10-366)

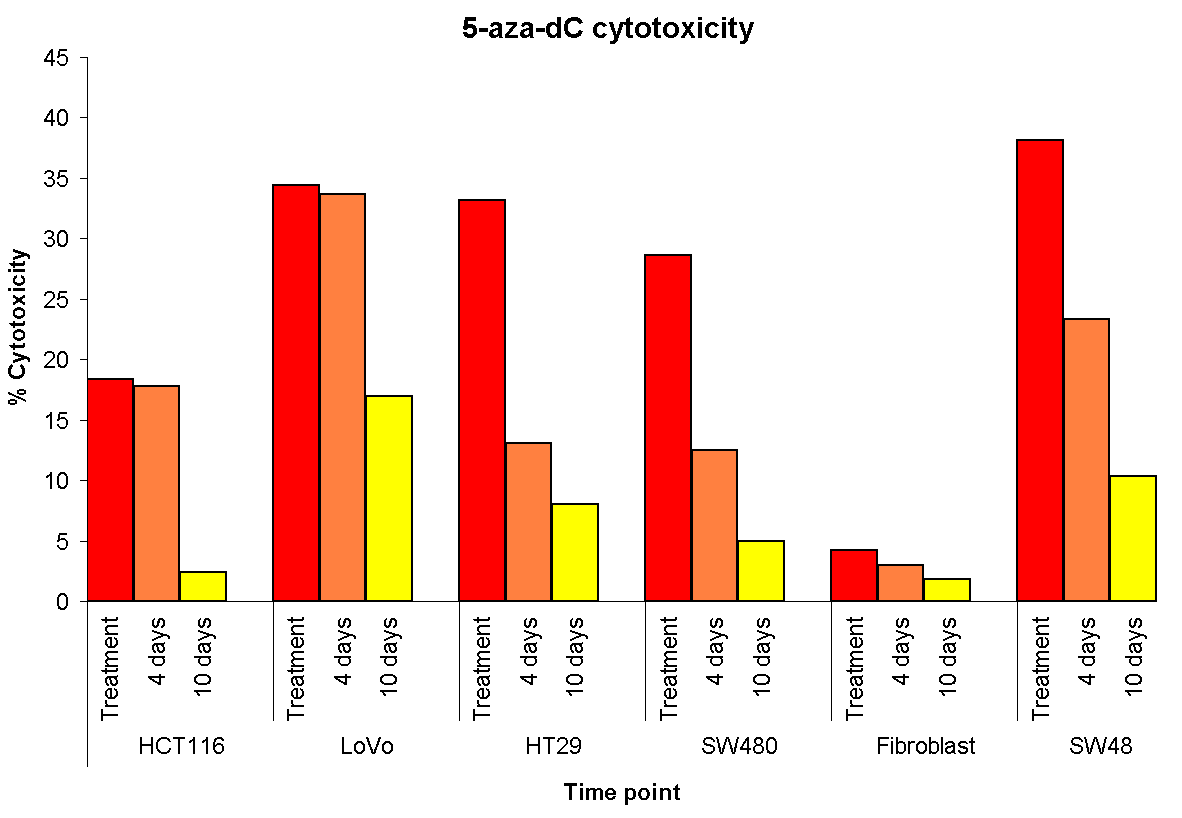

Supplement: Additional file 2 — Figure S2. 5-aza-dC induced cytotoxicity levels and recovery. Cytotoxicity was elevated immediately following treatment. By day 10 of the recovery period these levels had subsided to at least half of the initial value. [file 1471-2407-10-366-S2.TIFF]
